# The Dew Point

**Published:** 1856-01

**Authors:** James A. Kirkpatrick

**Affiliations:** Central High School, Philadelphia


### THE DEW POINT.

CENTRAL HIGH SCHOOL, *Philadelphia, Dec. 8th,* 1855.

*Mr. Editor*:—In answer to your inquiry in regard to the different means of observing and calculating the Dew Point, I would reply, that many instruments have been invented, by which the moisture existing in the atmosphere may be determined, as well as the reduction of temperature which will cause this moisture to be deposited upon any cold substance. The precise degree of temperature at which this deposition takes place, is called the dew point. The best instruments for indicating the dew point directly, are the hygrometers of Daniel, Regnault, and Bache. The two former, however, of these instruments have the defect of causing a great expense of ether, and not showing any deposition whatever, when the weather is very dry. *Bache's hygrometer* is more convenient, the requisite degree of coolness being produced by covering one end of a polished tube with pounded ice, or snow mixed with salt; while the temperature indicated by a very sensitive thermometer, placed at that part of the tube at which the moisture begins to precipitate, will be the dew point. The disadvantage attending the use of this instroment is, that it requires from twenty minutes to half an hour to make an observation; and in cold weather it is very difficult to obtain any indication of precipitation. *August's Psychrometer* is far preferable, and in all probability, will soon supersede all other *hygrometric* instruments, on account of its simplicity, and the facility with which it may be observed, and transported from place to place. It consists of two very sensitive thermometers, attached or freely suspended to the same frame; of the two bulbs, one is covered with a thin muslin or linen rag, and the other is left free. On moistening the rag, an evaporation takes place, which is more rapid in proportion as the degree of moisture in the air is small, or is farther removed from the point of saturation. This evaporation reduces the temperature of the covered bulb; so that from the difference of temperature indicated by the two thermometers, we may draw a conclusion in regard to the condition of moisture in the atmosphere. This instrument may be used when the temperature is below the freezing point, by covering one of the bulbs with a thin layer of ice. Sometimes, instead of dipping the covered bulb into water before every observation, it is kept constantly wet by the rag reaching down an inch or two, and dipping into a small vessel of water ; or sometimes a fountain is attached to the frame, like a common bird-cage fountain, into the open part of which the rag is dipped. It will thus keep constantly wet so long as there is any water in the fountain. Thus arranged, it is known as the *wet-bulb hygrometer*, or as *Mason's hygrometer*. This instrument, it will be perceived, does not give the dew point itself, but the point of greatest density; that is, the temperature at which the external air is so saturated with watery vapor, as to be incapable of taking up an additional amount, without condensation occurring with a further reduction. By the use of certain tables or formulæ, we may find the dew point from the indications of the psychometer. Unfortunately with this instrument, in its most convenient form, that of *Mason's hygrometer*, as sold by instrument makers generally, there is a printed table of instructions given, which professes to explain the method of finding the dew point, but which is very nearly right only for a temperature of about 40°. Below this point and above it, the results are far from correct.

The difficulties attending the exact determination of the dew point, and the fact, that when it is ascertained, it is in itself useless as a means of comparison, unless we are also acquainted with the temperature of the atmosphere at the same time, are gradually leading meteorologists to dispense with its observation or calculation altogether, and to substitute for it, the *force of vapor*, existing in the atmosphere, and the *relative humidity*, or the per centage of moisture when compared with complete saturation, both of which may be easily and accurately obtained from the indications of the *wet-bulb hygrometer*. When the first of these is ascertained, the dew point may be found from it at any time, as will appear hereafter.

Now, the greatest quantity of watery vapor that can exist in the atmosphere, at any particular temperature; and the corresponding *maximum tension* or *force*, or *pressure* which this quantity of vapor exerts upon a column of mercury, subject to the average atmospheric pressure, have been very accurately determined by experiments and observations often repeated and tested. From these experiments, it appears, that at the boiling point of water, 212°, the maximum tension of its vapor is equa[illegible]

to the atmospheric pressure, at the time and place of observation, and at the same time and place, the *maximum quantity* of vapor that can exist in one cubic foot of the atmosphere, is 256⅓ grains. In the same manner, the *maximum tension* and *quantity* of vapor, were ascertained by Dalton for every degree, and arranged in a table, for convenience of use and calculation. This table was afterwards corrected and extended, so as to give the tension and quantity of vapor for every degree, from — 31° to + 35° centigrade, by August of Berlin, and a formula constructed, by which the elastic tension or force of aqueous vapor, existing in the air at the time of observation might be calculated from it, and the indications of a psychrometer. In 1845, M. Regnault, in his *Etudes sur l'Hygrométrie*, published in the *Annales de Chimie et de Physique*, volume xv. of the 3d series, reviewed and corrected the tables given by Dalton and August, and also, by comparative experiments, slightly modified August's formulæ, reducing them to the values given in the September No. of the Examiner, in which Fahrenheit's degrees of temperature have been substituted for those of the centigrade thermometer, and English inches for millimetres.

The table of the *maximum tension* or *elastic force* of *acqueous vapor*, so far as it is necessary for meteorological calculations, as given by Regnault, but changed into the scale of Fahrenheit's thermometer and English inches, is given below, for whole degrees. For the fractions of degrees, the mean values may be adopted.

*Talle of the Maximum Force of Vapor of Water.*

| Temp. Fah. | Force of Vapor. | Temp. Fah. | Force of Vapor. | Temp. Fah. | Force of Vapor. | Temp. Fah. | Force of Vapor. | Temp. Fah. | Force of Vapor. |
|---|---|---|---|---|---|---|---|---|---|
| ° | Inches. | ° | Inches. | ° | Inches. | ° | Inches. | ° | Inches. |
| 1 | 0.0453 | 21 | 0.1128 | 41 | 0.2572 | 61 | 0.5367 | 81 | 1.0572 |
| 2 | 0.0473 | 22 | 0.1178 | 42 | 0.2672 | 62 | 0.5560 | 82 | 1.0922 |
| 3 | 0.0495 | 23 | 0.1233 | 43 | 0.2776 | 63 | 0.5758 | 83 | 1.1281 |
| 4 | 0.0520 | 24 | 0 1288 | 44 | 0.2884 | 64 | 0.5962 | 84 | 1.1651 |
| 5 | 0.0545 | 25 | 0.1346 | 45 | 0.2994 | 65 | 0.6174 | 85 | 1.2030 |
| 6 | 0.0570 | 26 | 0.1406 | 46 | 0.3109 | 66 | 0.6391 | 86 | 1.2421 |
| 7 | 0.0595 | 27 | 0.1466 | 47 | 0.3228 | 67 | 0.6616 | 87 | 1.2822 |
| 8 | 0.0623 | 28 | 0.1531 | 48 | 0.3351 | 68 | 0.6847 | 88 | 1 3235 |
| 9 | 0.0653 | 29 | 0.1596 | 49 | 0.3477 | 69 | 0.7085 | 89 | 1.3661 |
| 10 | 0.0683 | 30 | 0.1666 | 50 | 0.3608 | 70 | 0.7331 | 90 | 1.4096 |
| 11 | 0.0713 | 31 | 0.1736 | 51 | 0.3743 | 71 | 0.7585 | 91 | 1.4545 |
| 12 | 0.0747 | 32 | 0.1811 | 52 | 0.3883 | 72 | 0.7845 | 92 | 1.5006 |
| 13 | 0.0782 | 33 | 0.1884 | 53 | 0.4028 | 73 | 0.8114 | 93 | 1.5480 |
| 14 | 0.0818 | 34 | 0.1960 | 54 | 0.4177 | 74 | 0.8390 | 94 | 1.5967 |
| 15 | 0.0858 | 35 | 0.2040 | 55 | 0.4331 | 75 | 0.8676 | 95 | 1.6468 |
| 16 | 0.0898 | 36 | 0.2120 | 56 | 0.4491 | 76 | 0.8968 | 96 | 1.6981 |
| 17 | 0.0940 | 37 | 0.2205 | 57 | 0.4655 | 77 | 0.9272 | 97 | 1.7509 |
| 18 | 0.0985 | 38 | 0.2292 | 58 | 0.4824 | 78 | 0.9583 | 98 | 1.8051 |
| 19 | 0.1030 | 39 | 0.2382 | 59 | 0.4999 | 79 | 0.9903 | 99 | 1.8606 |
| 20 | 0.1078 | 40 | 0.2476 | 60 | 0.5180 | 80 | 1.0233 | 100 | 1.9178 |

The method of obtaining the *force of vapor* ($f$) at any time existing in the atmosphere, was fully explained in the Medical Examiner for September, 1855, and therefore need not be here repeated. It must suffice for the present to say a few words about the method of calculating the *Relative Humidity* and the *Dew Point.*

By *absolute moisture or humidity* we understand the quantity of water existing in the atmosphere in the state of vapor ; while by *relative humidity*, we mean the fraction which the *absolute moisture* existing, constitutes when compared with the maximum quantity, or the quantity necessary for saturation at the existing temperature. For example, a *relative humidity* of 0.45 means that the atmosphere contains $\frac{45}{100}$ of the quantity of vapor, which, at the temperature in question, would constitute saturation. Now the relative humidity is calculated by dividing the force of vapor existing in the air, as found by the formulæ above referred to, and before fully explained, by the maximum force of vapor for the temperature of the atmosphere. Thus, suppose the *dry bulb* of the psychrometer indicates 58°, at the same time that the *wet bulb* thermometer is found to mark 54°. Then by the formula

$$f = f' - \frac{0.480\,(t-t')}{1130-t'}\,h.$$

and making ($h$) the height of the barometer 29.92, its mean height at Philadelphia, we find that the force of vapor ($f$) = 0.3643 ; then dividing this by the maximum force of vapor for 58°, which, by the table on the preceding page, is seen to be 0.4824, we will have 0.755 for the relative humidity, showing that the air conains $\frac{75}{100}$ or $\frac{755}{1000}$ of the quantity of vapor which at the same temperature, 58°, would be necessay to complete saturation.

It is evident from the method of finding the relative humidity just explained, that the force of vapor ($f$) existing in the air, must be the maximum force for the temperature of the dew point. Therefore, in order to find the dew point corresponding to the indications of the psychrometer before given, that is 58° for the dry bulb, and 54° for the wet bulb, we have only to look in the table of Maximum Force of Vapor for the temperature corresponding to force ($f$) existing in the atmosphere ; or in other words, the force of vapor existing in the air at the time of observation, is the same as the *Maximum Force of Vapor*, for the temperature of the dew point at the same time. By referring to the table, it will be seen that the temperature at which the force of vapor (0.3643) is the maximum force, is between 50 and 51 degrees, or 50.3°, which must consequently be the dew point, when the indications of the psychrometer and barometer are such as we have supposed.

Proceeding in this way, I have re-calculated the dew point for each month from the beginning of my Meteorological Observations, and find it to be as follows, at Philadelphia for 2 P. M.

| | 1851 | 1852 | 1853 | 1854 | 1855 |
|---|---|---|---|---|---|
| January, | | 30.50 | 27.02 | 30.62 | 28.60 |
| February, | | 28.30 | 34.45 | 31.64 | 18.76 |
| March, | | 37.50 | 31.05 | 34.93 | 27.31 |
| April, | | 37.10 | 42.86 | 40.79 | 40.45 |
| May, | | 53.20 | 53.65 | 53.59 | 46.08 |
| June, | | 59.00 | 63.17 | 62.92 | 59.37 |
| July, | 65.83 | 64.50 | 65.58 | 68.52 | 68.66 |
| August, | 64.59 | 62 90 | 66.95 | 65.30 | 64.03 |
| September, | 58.41 | 54.80 | 60 45 | 61.24 | 60.72 |
| October, | 50.40 | 51.60 | 42.31 | 50.89 | 47.02 |
| November, | 35.12 | 35.00 | 40.92 | 37.82 | 39.40 |
| December, | 30.61 | 37.70 | 28.33 | 26.56 | |
| Mean annual Dew Point, | | 46.01 | 46.40 | 47.07 | |

To lessen as much as possible the complicated calculations, which I have explained, I have constructed a table which gives, from the temperature ($t'$) of the wet-bulb thermometer, and the temperature ($t$) of the dry-bulb or their difference ($t—t'$) the *Force of Vapor* in the atmosphere, the *Relative Humidity* and the *Dew Point*, for every degree of temperature, from—19° to 100°, Fahrenheit, supposing the barometer to remain at the same average stand of 29.92 inches. It is evident that every observer, by calculating such a table for himself, and, substituting in the formula for 29.92 ($h$) the mean height of the barometric column at his own station, may save considerable time and labor, for when the numbers are once found, they never require to be re-calculated for the same degrees. If it should be deemed necessary to make a correction for the different heights of the barometer, a table may also be constructed for this purpose. This, however, is not necessary for ordinary meteorological purposes.

James A. Kirkpatrick, A. M.

